# Life lies in movement

**DOI:** 10.1093/lifemedi/lnaf024

**Published:** 2025-07-02

**Authors:** Junzhu Yi, Yuanyuan Du

**Affiliations:** Hangzhou Institute of Medicine (HIM), Chinese Academy of Sciences, Hangzhou 310000, China; Hangzhou Institute of Medicine (HIM), Chinese Academy of Sciences, Hangzhou 310000, China

The benefits of regular exercise for physical and mental health are well-known. However, how exercise exerts its health effects at the cellular and molecular levels remains unclear. Recently, a study published in *Cell* provides a comprehensive analysis of the mechanisms underlying the health benefits of exercise through multi-omics profiling in humans and mouse models [1]. By integrating single-cell transcriptomics, plasma proteomics, metabolomics, and fecal microbiome analysis, the study systematically deciphered the mechanisms by which exercise delays aging at the cellular and molecular levels.

The study first enrolled 13 healthy young males and designed a three-phase intervention: a 45-day baseline, a single acute exercise session, and a 25-day long-term exercise session, coupled with longitudinal sampling of blood, stool, and physiological parameters. They observed distinct molecular response patterns induced by acute and long-term exercise. Acute exercise triggered transient metabolic stress marked by surging non-esterified fatty acids and inflammatory cytokines reflecting a “fight-or-flight” response. In contrast, long-term exercise induced profound adaptive reprogramming, marked by significant reductions in indirect bilirubin, gamma-glutamyl transferase, triglycerides, and inflammatory markers such as C-C motif chemokine ligand 25 (CCL25), high-sensitivity C-reactive protein, and tumor necrosis factor-α. Long-term exercise was further associated with physiological improvements, including reduced body mass index and a lower resting heart rate.

Interestingly, the authors uncovered a strong association between long-term exercise and immune cell remodeling. Long-term exercise induced increasing of lymphocytes, especially all naïve lymphocyte populations, and a decrease in myeloid cells, particularly neutrophils. The elevated expression of Coronin 1a (CORO1A) and Cluster of differentiation 74 (CD74) indicated the enhanced active immune cell migration and mobilization capability by long-term exercise. Furthermore, peripheral blood cells from individuals engaging in long-term exercise showed the enhancement of heterochromatin marker histone H3 lysine 9 trimethylation (H3K9me3), phosphorylated nuclear factor erythroid-2-related factor 2 (NRF2), and upregulated expression of NRF2-responsive antioxidant genes (*TXN*, *GPX1*, *SOD1*, and *GSTP1*), indicating a robust enhancement of cellular antioxidant capacity. Collectively, these results suggested that long-term exercise reprogrammed the immune system toward a youthful state.

The study also unraveled the correlation between long-term exercise and metabolism, and highlighted the crucial role of betaine, the endogenous metabolite elevated by sustained exercise, in geroprotective effects. The author found a robust increase in activated methionine metabolism and amino acid metabolism, particularly betaine metabolism after long-term exercise. In mice undergoing two-week treadmill endurance training, the levels of betaine in many tissues were upregulated, especially in the kidney. Consistently, the expression of choline dehydrogenase, a key enzyme involved in choline-to-betaine conversion also increased. Single-nucleus RNA sequencing in mouse kidneys further revealed that betaine could mitigate aging signatures in most cell types, particularly in renal tubular epithelial cells. Circadian genes that decreased with aging were restored after betaine intervention, while increased inflammatory genes were suppressed.

**Figure 1.**
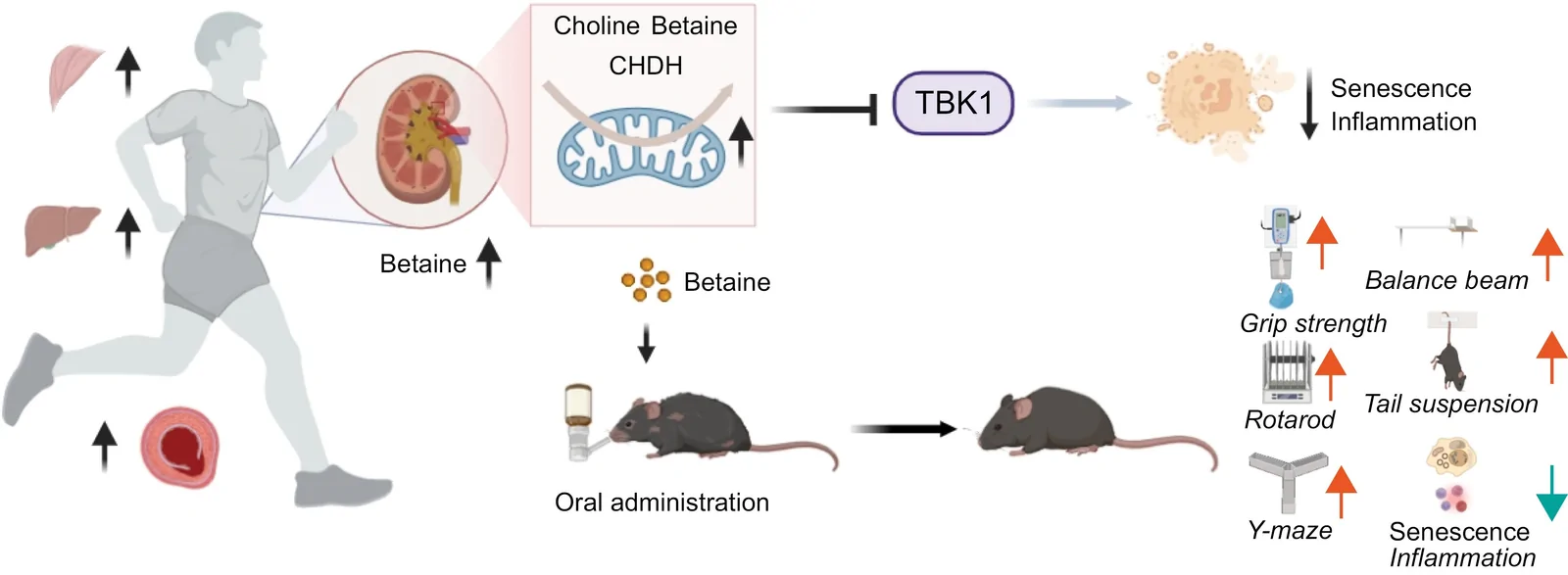
Betaine attenuating senescence and inflammation in human and mouse.

Notably, using mouse model, the author showed that oral administration of betaine (0.1%–1% *w*/*v*) significantly reduced the senescence markers across multiple organs, while physical function, cognitive performance, and metabolic parameters were greatly improved (Fig. 1). Previous studies showed that betaine reduces homocysteine concentrations, potentially mitigating vascular, neurodegenerative, and oxidative damage [2]. Betaine has also been shown to enhance mitochondrial function, reduce oxidative stress, and attenuate inflammation [2]. Considering the biosafety and oral bioavailability, these studies suggested the clinical translation potential of betaine, especially for aging individuals with limited exercise capacity.

Mechanistically, betaine bond to and inhibited TANK-binding kinase 1 (TBK1) (Fig. 1). Studies have shown that TBK1 drove aging through reactivation of endogenous retroviruses, mitochondrial DNA leakage, and the silencing of desuccinylase Sirtuin 5 (SIRT5) [3, 4]. TBK1 also played a role in age-related diseases like renal fibrosis and neurodegenerative conditions [5, 6, 7]. Amlexanox, an Food and Drug Administration (FDA)-approved TBK1 inhibitor, has shown therapeutic effects against renal fibrosis in rodents [7]. These findings suggested betaine as an exercise mimetic for healthy aging.

In summary, by synergistically integrating multi-layered analysis, this study demonstrated that long-term exercise induced comprehensive cellular and molecular adaptations, establishing a mechanistic link between physical activity and molecular regulatory networks underlying healthy longevity. The identification of betaine as a key metabolite involved in exercise-induced benefits paves a new way to develop “exercise in a pill” approaches. The study’s findings not only deepen our understanding of exercise physiology but also offer potential targeted intervention strategies to promote healthy aging.
